# Seronegative Coeliac Disease in Children: A Case Report and Review of the Literature

**DOI:** 10.1155/2017/1652052

**Published:** 2017-03-13

**Authors:** Vinod Kolimarala, Ekta Vasita, Hany Banoub, Sonny K. F. Chong

**Affiliations:** Epsom and St. Helier University Hospitals NHS Trust, Carshalton, UK

## Abstract

Serology is frequently used for the diagnosis of coeliac disease in children; however, a small proportion of children are seronegative. We present a case of seronegative coeliac disease along with literature review to include diagnostic and management dilemmas.

## 1. Introduction

Coeliac disease (CD) is a gluten-dependent autoimmune systemic disease, presenting in individuals with a genetic predisposition, namely, HLA-DQ2 and HLA-DQ8 haplotypes [1]. The estimated prevalence of CD in the UK is 1 : 100 [1]; however, it is currently being underdiagnosed, with as many as 90% of diagnoses being missed [1, 2].

Symptoms include abdominal pain, diarrhoea, faltering growth, idiopathic short stature, vomiting, abdominal distention, constipation, dermatitis herpetiformis, dental enamel defect, osteoporosis, pathological fractures, delayed menarche, unexplained anaemia or treatment-resistant iron-deficiency anaemia, recurrent aphthous stomatitis, unexplained hepatic disease, and weakness.

Serological testing is used by many clinicians due to reliability and accessibility. Many clinicians believe the presence of a normal IgA and normal tTG-IgA rules out coeliac disease due to high sensitivity of the test and do not investigate further. Here we present the case report of a teenager with seronegative coeliac disease (SNCD) and literature review to highlight the diagnostic and follow-up management difficulties.

## 2. Case Report

A 14-year-old boy was referred by his General Practitioner with history of abdominal pain and rash on his back for 12 months. The rash was treated with multiple topical agents including antifungal, antibiotic, and steroid ointments with no improvement. There was no history of loss of appetite, loose stools, diarrhoea, or bloating. He had no significant past medical history or medical problems within the family. He was weaned to solids without any difficulties and had no known food allergies.

On examination, he had a maculopapular and urticarial rash on his back and knees. The rest of his examination was normal.

Investigations including FBC, LFT, U&E, ESR, CRP, anti-tTG, IgA, IgG, and IgM all were normal, but anti-endomysial antibody was weakly positive.

Upper GI endoscopy showed normal oesophagus, mild antral erythema (negative CLO test), and normal mucosa in the duodenum. Partial villous atrophy with crypt lengthening and increasing intraepithelial lymphocytes was seen on histology. A diagnosis of seronegative CD was made. Quick mucosal lactase test was mildly suggestive of lactose intolerance.

The patient noticed a significant improvement in his rash within 12 days of starting gluten-free diet (GFD). He continued to improve on a GFD and when seen in the follow-up clinic a year later reported complete resolution of symptoms. His weight improved from 66.5 kg to 75 kg and height improved from 179.3 cm to 183.6 cm. Repeat investigations including anti-tTG antibody were normal. As there was improvement in his clinical symptoms, height, and weight, we did repeat endoscopy or do HLA-DQ2 and HLA-DQ8 investigations.

## 3. Discussion

Diagnosis and long-term follow-up management of SNCD remain a challenge. Investigations for CD mainly include anti-tTG antibodies, IgA, endoscopy, and histopathology. We present both advantages and limitations of serological testing, histopathological diagnoses, and follow-up management:

1. Regarding serological testing, currently there is a lot of reliance on serological testing for coeliac disease following the publication of BSPGHAN and Coeliac UK guidelines. The guidelines suggest that, in symptomatic children, CD is unlikely if anti-tTG antibodies are negative provided IgA is normal. If clinically CD is still suspected, then an endoscopy with duodenal biopsies should be performed [1]. The sensitivity and specificity of anti-tTG-IgA antibody are reported between 81 and 100% and between 97 and 99% [3], respectively. It is of interest that a negative predictive value of 99.6% would infer that a negative serology test would essentially allow a clinician to rule out the diagnosis of CD.
2. Sensitivity and specificity of anti-EMA antibody are between 74 and 100% and between 99 and 100%, respectively [4]. The sensitivity and specificity of serology testing largely depend on the investigating laboratory [5]. 150 blood samples were given blindly to 15 laboratories; the sensitivities ranged from 62 to 92%. In the absence of standardisation, reliance on serological testing is questionable.
3. Tissue damage typical of CD is characterized by villous atrophy and crypt hyperplasia. Villous atrophy can be the result of other gastrointestinal conditions like parasitic infections, autoimmune conditions affecting the bowel, small bowel bacterial overgrowth, and Crohn's disease [6]. Reversal of symptoms and abnormal histology after a GFD for a suitable period of time would confirm a diagnosis of SNCD. Murch et al. suggest gluten rechallenge to confirm uncertain diagnoses with HLA-DQ2 and HLA-DQ8 tests; negative HLA test renders CD diagnosis unlikely. This emphasises the difficulties encountered when considering diagnoses in a clinical setting.
4. As for monitoring of patient, current guidelines recommend monitoring by a paediatric dietician, a paediatric gastroenterologist, or paediatrician with an interest in gastroenterology to allow for long-term adherence [1]. Follow-up of 6–12 months after the introduction of GFD should include symptoms review, growth and full physical examination, assessment of micronutrient levels, and anti-tTG antibody testing. Thereafter, assessment of disease status is monitored by anti-tTG antibody titres, which presents a problem in patients with SNCD. Apart from symptom resolution and blood tests investigating micronutrient status, how else can SNCD patients be best monitored continues to remain an unanswered question.

## 4. Conclusion 

SNCD is a disease that requires significant recognition as consequences of misdiagnosis lead to increased morbidity and mortality. Negative serology tests must not lead to the dismissal of a coeliac disease diagnosis. There is a need for more definitive diagnostic and management guidelines.
